# Variations in oral microbiome profiles in rheumatoid arthritis and osteoarthritis with potential biomarkers for arthritis screening

**DOI:** 10.1038/s41598-018-35473-6

**Published:** 2018-11-20

**Authors:** Bin Chen, Yan Zhao, Shufeng Li, Lanxiu Yang, Haiying Wang, Tao Wang, Bin Shi, Zhongtao Gai, Xueyuan Heng, Chunling Zhang, Junjie Yang, Lei Zhang

**Affiliations:** 1 College of Life Science, Shandong Normal University, Jinan, 250014 China; 2 Shandong Medicinal and Biotechnology Centre, Shandong Academy of Medical Sciences, Jinan, 250062 China; 3 Department of Orthopedics, Qianfoshan Hospital Affiliated to Shandong University, Jinan, 250014 China; 4 Guoyitang Hospital, Jinan, 250000 China; 5 College of Life Science, Qilu Normal University, Jinan, 250200 China; 6 Shandong Institutes for Food and Drug Control, Jinan, 250101 China; 7 Qingdao Human Microbiome Center, The Affiliated Central Hospital of Qingdao University, Siliu South Road 127, Qingdao, Shandong Province 266042 China; 8 Shandong Children’s Microbiome Center, Qilu Children’s Hospital of Shandong University, Jinan, Shandong Province 250022 China; 9 Microbiological Laboratory, Department of Infection Management, Department of Neurosurgery, Lin Yi People’s Hospital, Linyi, Shandong Province 276003 China; 10 Beijing Advanced Innovation Center for Big Data-Based Precision Medicine, School of Chemistry and Environment, Beihang University, Beijing, 100191 China

## Abstract

The key to arthritis management is early diagnosis and treatment to prevent further joint destruction and maximize functional ability. Osteoarthritis (OA) and rheumatoid arthritis (RA) are two common types of arthritis that the primary care provider must differentiate, in terms of diagnosis and treatment. Effective and non-invasive strategies for early detection and disease identification are sorely needed. Growing evidence suggests that RA has a correlation with oral microbiome and may be affected by its dynamic variations. There is already a study comparing oral microbiome in patients with RA and OA, however, it did not screen for potential biomarkers for arthritis. In this study, we assessed the oral microbiome in saliva samples from 110 RA patients, 67 OA patients and 155 healthy subjects, using 16S rRNA gene amplicon sequencing. The structure and differences in oral microbiome between RA, OA and healthy subjects were analyzed. Eight oral bacterial biomarkers were identified to differentiate RA from OA. This report provides proof of oral microbiota as an informative source for discovering non-invasive biomarkers for arthritis screening.

## Introduction

Rheumatoid arthritis (RA) is a highly prevalent, chronic inflammatory and systemic autoimmune disease. Multiple clinical studies have suggested a potential link between RA and periodontopathic bacteria such as *Porphyromonas gingivalis*^1^. Traditionally, osteoarthritis (OA) is viewed as a mechanically induced condition, and the role of inflammatory factors, such as those derived from microbiota, has been considered minor. Although there are significant differences in the pathogenesis of RA and OA, the clinical symptoms of rheumatoid arthritis and osteoarthritis are similar and sometimes difficult to distinguish, especially in the case of unilateral knee RA, which is more difficult to distinguish from OA. It may also affect prognosis in patients. Therefore, it is necessary to find a strategy for early detection of arthritis.

Recent molecular investigations have indicated that each individual carries over 200 species in their oral microbiome^2^. The relationship between periodontopathic bacteria and systemic diseases has been explored for several years^3–5^. Several studies have confirmed that oral bacteria have been associated with pancreatic cancer^6^, Parkinson’s disease^7^, systemic lupus erythematosus^8^, colorectal cancer, lung inflammation^9^, type–2 diabetes^10,11^. Oral bacteria can be mobile and affect sites in and around the oral cavity. It can enter the bloodstream easily and also during daily mastication, brushing, flossing and in diseased state. Oral bacteria may penetrate through relatively permeable epithelial pockets to reach the underlying gingival connective tissue, enter the bloodstream and travel throughout the body^12,13^.

Many clinical studies have shown that RA and OA may be associated with oral microbiota and are affected by it. Scher *et al*.^14^ used pyrosequencing to compare the composition of subgingival microbiota in patients with RA and controls, revealing that *Prevotella* and *Leptotrichia* species only existed in RA. Subsequently, a more intensive study performed metagenomic shotgun sequencing to detect the dental and salivary microbiome in patients with RA and controls. It was observed that *Haemophilus* spp. decreased in RA, and *Lactobacillus salivarius* numbers increased. Also, the functions of oral microbiome changed in RA^15^. However, recent findings suggest that OA may also be regulated by the microbiome, but there are very few studies describing the potential effects of oral microbiota in patients with OA.

In our study, we aim to describe the oral microbiome in patients with RA, OA and healthy subjects, and their potential association with RA and OA. We also attempted to distinguish RA and OA by identifying differences in oral microbiome and found a set of potential biomarkers for distinguishing RA and OA non-invasively.

## Results

### Variations in Oral Microbiota Profiles in RA and OA

**Figure 1:**
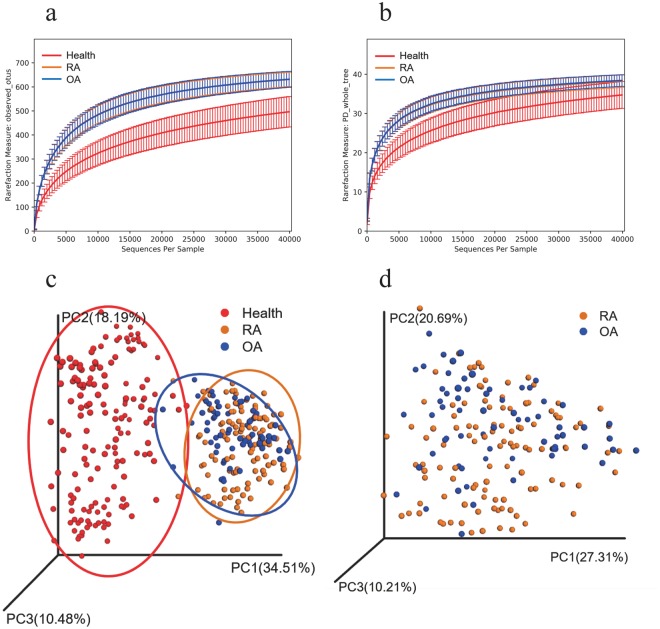
Alpha and beta diversity in RA, OA and healthy subjects. (**a**) Rarefaction analysis of observed OTUs. Using the mean of observed OTUs randomly sampled 150 times at 40000 sequencing depth. Error bars represent standard deviation. (**b**) Rarefaction analysis of Shannon index. Using the mean of observed OTUs randomly sampled 150 times at 40000 sequencing depth. Error bars represent standard deviation. (**c**) Weighted UniFrac principle coordinate analysis of RA, OA and healthy subjects. Ellipses are added to better visualize the cluster and separation between RA, OA and healthy control. (**d**) Weighted UniFrac principle coordinate analysis of RA and OA.

**Figure 2:**
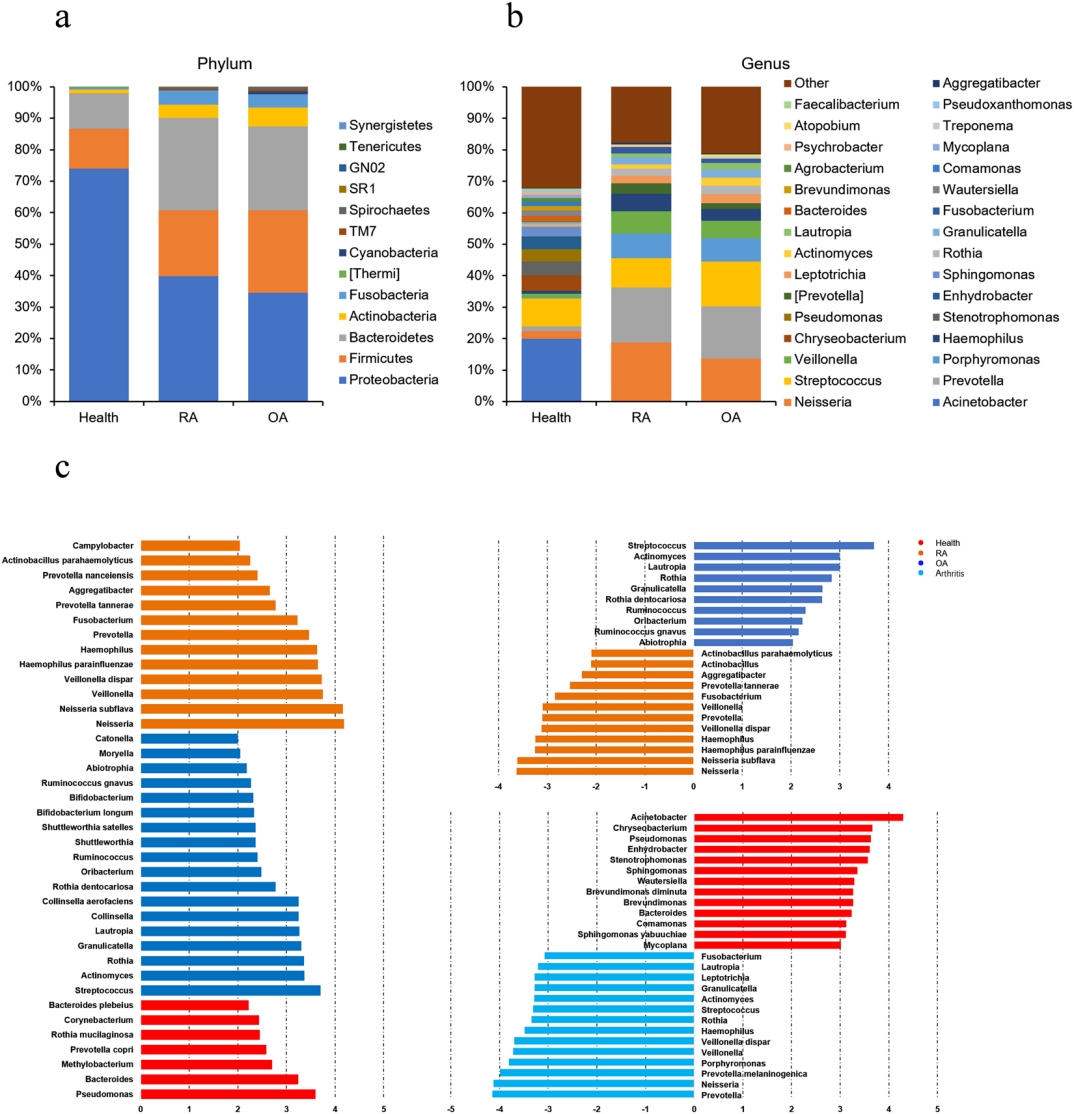
Taxonomic profiles and biomarkers of patients with RA, OA and healthy subjects. (**a**) Barplots of taxonomic profiles of patients with RA, OA and healthy subjects at the Phylum level. (**b**) Barplots of taxonomic profiles of patients with RA, OA and healthy subjects at the genus level. (**c**) Histogram of the LDA scores, where the LDA score indicates the effective size and ranking of each differentially abundant taxon (LDA > 2).

We compared the oral microbiota profiles of 110 RA patients, 67 OA patients and 155 healthy subjects. Alpha diversity analysis reveals that there was no significant difference in observed operational taxonomic units (OTUs) (*P* = 0.213, Welch’s t-test, Fig. 1a) and PD whole tree index (*P* = 0.426, Welch’s t-test, Fig. 1b) between RA and OA, while the healthy group had significant differences compared to groups with RA (*P* < 0.001) and OA (*P* < 0.001). To visualize the overall differences in beta diversity between the microbiome profiles of these three groups, we conducted a Principal Coordinate Analysis (PCoA) of weighted and unweighted UniFrac distances. Beta diversity comparisons using Analysis of similarities (ANOSIM) for oral data suggested a minor, but statistically significant community difference between RA and OA (ANOSIM, R = 0.07, *P* = 0.001) (Fig. 1c,d). The data in healthy subjects suggested significant differences from that of RA and OA. The microbial composition of RA, OA and healthy subjects did differ at the phylum and genus levels (Fig. 2a,b). The most common phyla were Proteobacteria, Firmicutes, Bacteroidetes, Actinobacteria and Fusobacteria. The relative abundance of Proteobacteria in healthy subjects was significantly higher than in patients with RA and OA (*P* < 0.001, Welch’s t-test), and the relative abundance of Firmicutes (*P* < 0.029, Welch’s t-test) in patients with OA is significantly higher than those in patients with RA. To further evaluate microbiome differences among patients with RA, OA and healthy subjects, the Linear discriminant analysis (LDA) Effect Size (LEfSe) method was used to discover the difference in microbiota composition and identify significant biomarkers, thus revealing 24 genera and 14 species (LDA > 2) (Fig. 2c). Many of the taxa were differentially abundant in RA and OA and contained: *Neisseria subflava*, *Haemophilus parainfluenzae*, *Veillonella dispar*, *Prevotella tannerae*, *Actinobacillus parahaemolyticus*, *Neisseria*, *Haemophilus*, *Prevotella*, *Veillonella*, *Fusobacterium*, *Aggregatibacter*, and *Actinobacillus*, which were more in RA, while *Rothia dentocariosa*, *Ruminococcus gnavus*, *Streptococcus*, *Actinomyces*, *Lautropia*, *Rothia*, *Granulicatella*, *Ruminococcus*, *Oribacterium*, and *Abiotrophia* numbers increased in OA (Fig. 2c). We also compared healthy subjects with the arthritis group (RA + OA), revealing potential opportunistic oral pathogens. *Prevotella melaninogenica*, *Veillonella dispar*, *Prevotella*, *Neisseria*, *Porphyromonas*, *Veillonella*, *Haemophilus*, *Rothia*, *Streptococcus*, *Actinomyces*, *Granulicatella*, *Leptotrichia*, *Lautropia*, and *Fusobacterium* were increased in the arthritis group (Fig. 2c). Since gender and age varied significantly among the groups, it may confound the identified associations. We tested the age and gender effects on the oral microbiome using Spearman’s rank correlation test (Supplemental Table S2). The results indicate that oral microbiome has no significant correction with gender. There are only 5 taxonomies affected by the factor of age, however, they don’t belong to the identified different abundant taxonomies among RA, OA and healthy subjects.

### Imputed functions of Oral Microbiota in RA and OA

**Figure 3:**
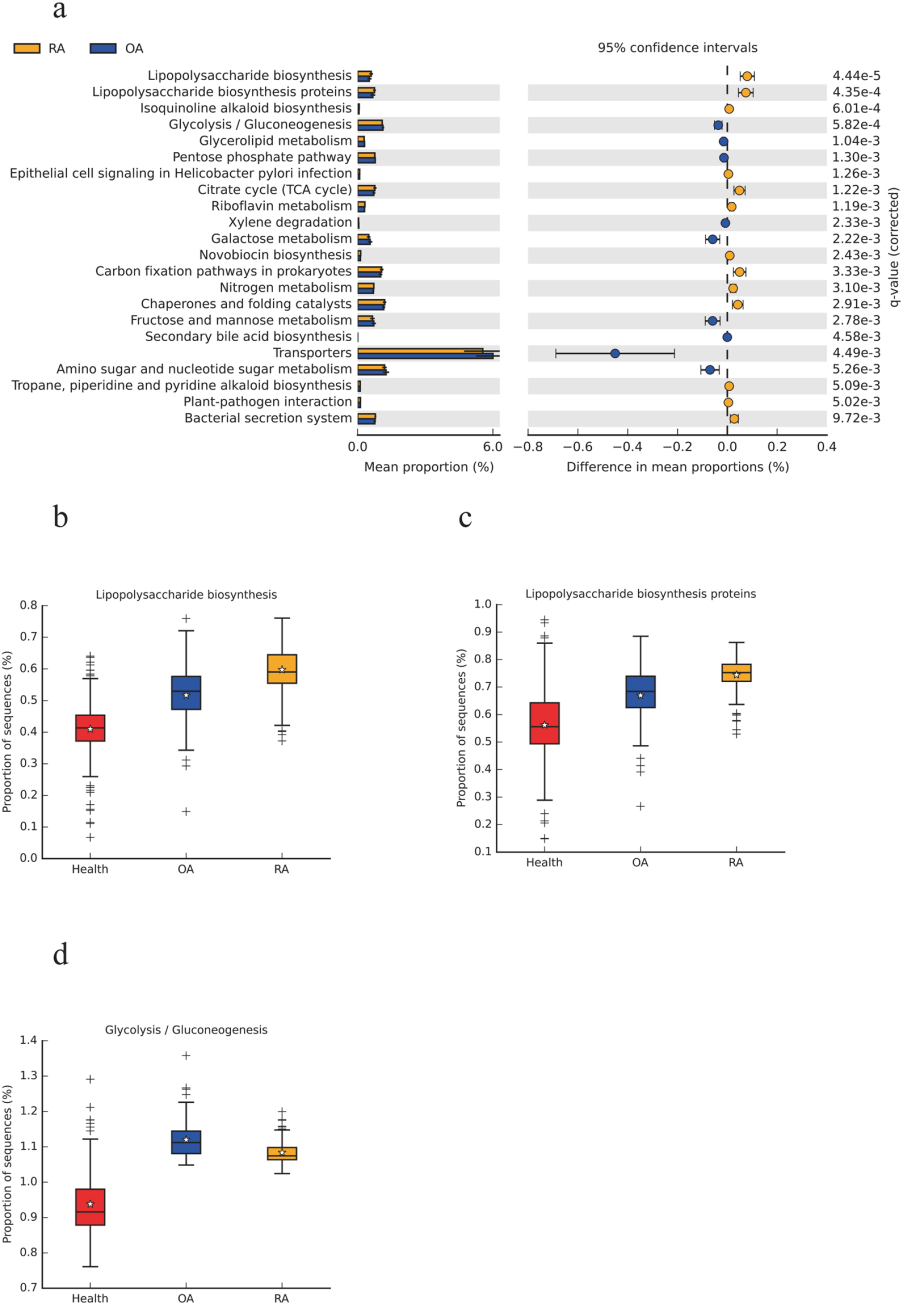
Functional analysis of oral microbiota in patients with RA, OA and healthy subjects. (**a**) Extended error barplot with 95% confidence intervals showing significantly different KEGG pathways between RA and OA. Corrected P-values are calculated using Benjamini-Hochberg FDR approach (**b**) Lipopolysaccharide biosynthesis (**c**) Lipopolysaccharide biosynthesis proteins (**d**) Glycolysis/Gluconeogenesis; *indicates the mean of the data, the data points outside of the whiskers are shown as crosses+.

Given the structural changes within the oral microbiota in patients with RA and OA, we explored microbiota functions based on inferred metagenomes using the Phylogenetic Investigation of Communities by Reconstruction of Unobserved States (PICRUSt) algorithm. PICRUSt is a computational approach in which evolutionary modeling is used to predict the present gene families from 16S rRNA gene sequencing data and a reference genome database. In all, 6,909 predicted genes were assigned to 301 KEGG, level 3 modules. Forty-seven pathways were significantly different between RA and OA (*P* < 0.05; Welch’s t-test, Supplemental Table S3), of which 28 KEGG pathways were related to metabolism. The differences between 22 pathways were extremely significant between RA and OA (*P* < 0.01; Welch’s t-test, Fig. 3a). The most significantly different KEGG pathways included lipopolysaccharide biosynthesis (Fig. 2b), lipopolysaccharide biosynthesis proteins (Fig. 3c) and glycolysis/gluconeogenesis (Fig. 3d). For KEGG hierarchical level 1 functional modules, metabolism was the most abundant module, then genetic information processing and environmental information processing (Supplemental Fig. S1). The signaling and environmental information processes were significantly different between RA and OA (*P* < 0.05, Welch’s t-test). For KEGG hierarchical level 3 functional modules, transporters were the most abundant module, followed by ABC transporters and DNA repair, then recombination proteins (Supplemental Fig. S2).

### Potential biomarkers for distinguishing RA and OA

**Figure 4:**
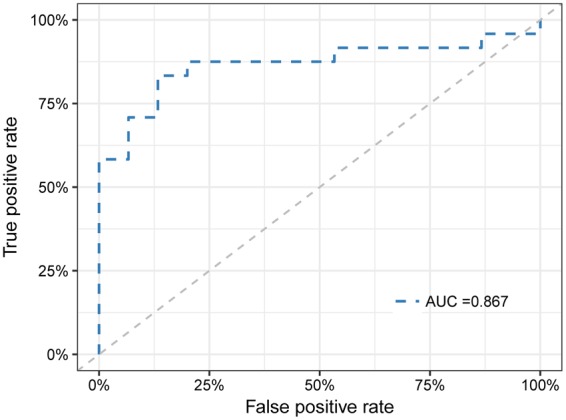
The ROC curve based on 8 most-distinctive OTUs. The maximum AUC value is then selected and drawn.

To evaluate the potential value of identified bacterial biomarkers for clinical differentiation of RA and OA, we constructed the receiver operating characteristic (ROC) curve and computed the area under the curve (AUC) values. We chose 10 OTUs with an LDA value greater than 3.0 in the LEfSe analysis, as candidate biomarkers (*Streptococcus*, *Actinomyces*, *Lautropia*, *Neisseria*, *Neisseria subflava*, *Haemophilus parainfluenzae*, *Haemophilus*, *Veillonella dispar*, *Prevotella* and *Veillonella*). To verify the best prediction model, we constructed the ROC curve for each arrangement combination in these 10 candidate biomarkers, as the prediction model. The training and validation sets were randomly selected 5 times and the average AUC was calculated (Supplemental Table S4). The average AUCs of 3 biomarker models were greater than 0.85 (Fig. 4). Moreover, the biomarker model with 8 OTUs (*Actinomyces*, *Neisseria*, *Neisseria subflava*, *Haemophilus parainfluenzae*, *Haemophilus*, *Veillonella dispar*, *Prevotella* and *Veillonella*) generated the highest AUC value of 0.8756.

## Discussions

We compared the oral microbiota in patients with RA, OA and healthy subjects. We showed that profiling the bacteria associated with the oral cavity may have value in the detection of RA and OA. We also found that patients with RA and OA had an oral microbiota with higher microbial diversity compared to healthy subjects, indicating that there could be more harmful bacteria or opportunistic pathogens in the oral cavity of patients with RA and OA. Interestingly, when compared to healthy subjects, the following potential opportunistic oral pathogens increased in the arthritis group (RA + OA): *Prevotella melaninogenica*, *Veillonella dispar*, *Prevotella*, *Neisseria*, *Porphyromonas*, *Veillonella*, *Haemophilus*, *Rothia*, *Streptococcus*, *Actinomyces*, *Granulicatella*, *Leptotrichia*, *Lautropia*, and *Fusobacterium*. Most of them are Gram-negative anaerobic bacteria. In keeping with this, *Porphyromonas, Prevotella melaninogenica, Actinomyces* and *Streptococcus* are considered to be directly responsible for periodontitis^16^. Previous studies also reported that they were associated with RA^1,17,18^. *Porphyromonas gingivalis* has been demonstrated to be directly linked to RA through citrullination and induction of antipeptidyl citrulline antibodies reacting to citrullinated human self-proteins^19^. *P. melaninogenica* causes a deficiency of terminal galactose residues by binding to the Fc region of the IgG molecule and metabolizing galactose with its enzymes^20^. A lack of terminal galactose residues early in the course of RA is associated with a worsening prognosis^21^. In patients with chronic inflammatory diseases, oral microbiome dysbiosis is associated with the disease. Previous studies have found that alpha diversity of oral microbiome was significantly decreased in Sjögren syndrome^22^ and Behçet’s disease^11^, but our study found an increased alpha diversity in the patients, which could be due to the difference of disease. Consistent to these studies, our study also found an increase in the abundance of *Streptococcus* and *Haemophilus* in the diseased subjects.

Very few studies have been conducted on oral microbiome of patients with RA and OA using next-generation sequencing (NGS). Mikuls *et al*.^23^ compared the subgingival microbiome of RA and OA patients. Compared to OA, 10 taxa were lower in numbers in patients with RA, and this study failed to identify a biomarker that could reliably distinguish RA from OA. The result from our study contrasted with the report from Mikuls *et al*., and only *Streptococcus* was consistent. We found that *Prevotella* was more abundant in patients with RA instead of being lower. Scher *et al*.^14^ also revealed that *Prevotella was found* in greater abundance in patients with RA. Differences in the findings across studies may relate to the oral sampling site. Our study collected saliva samples instead of subgingival plaque like in Mikuls *et al*. The research indicated that the microbiome between supragingival and salivary communities displayed strong differences^24,25^. We also used machine learning to conduct a classification analysis for selected biomarkers. We succeeded in identifying biomarkers that could reliably distinguish RA from OA in patients.

The translocation of microbiota-derived molecules into the systemic circulation is one route for the microbiome to mediate osteoarthritis. A recent study reported that systemic and synovial concentrations of bacterial Lipopolysaccharide (LPS) positively correlated with joint inflammatory response and the severity of joint degradation^26^. LPS also plays a role in RA and can upregulate all of the cytokines^27^. Furthermore, bacteria-derived LPS by itself is also capable of potentiating collagen-induced arthritis (CIA) in mice. In a nutshell, oral bacteria or bacteria-derived LPS could invade blood and be transported to joints, where they could stimulate innate immune receptors in bone, cartilage and synovium. Lyu^28^ *et al*. detected the key drivers in oral microbial community related to dysbiosis in RA. They found that the development of biofilms is one of the drivers of persistent infections. LPS was found to play a role in biofilm formation^29^, and previous study also found that *P. gingivalis* can grow in the biofilm, which can become destructive and may contribute to RA^30^.

Despite significant advances in understanding pathophysiology of RA and OA, early diagnosis and therapeutic intervention remain a challenge^31^. Rheumatoid factor (RF) and cyclic citrullinated peptides (CCPs) have usually been used as the biomarkers for RA, but have relatively low sensitivity^32^ and low specificity^33^ for early RA. Magnetic resonance imaging (MRI) techniques have been developed for early-stage evaluation of cartilage damage in OA^34^, however, MRI is expensive and not easily/widely accessible. There are a host of advantages in using oral microbiota as a source of biomarkers for predicting and screening disease. Saliva is a non-invasive collection method that does not cause discomfort and pain to subjects. Therefore, it is widely used in the study of biomarkers in diseases. We profiled the oral microbiome and discovered 8 biomarkers that offered an alternative strategy for the diagnosis of RA and OA. Further studies to validate these potential biomarkers using a larger clinical cohort are warranted.

## Conclusions

We explored the differences in oral microbiome profiles of patients with RA, OA and healthy subjects using next-generation sequencing. Most importantly, we compared the oral microbiome and their potential functions in RA, OA and healthy subjects. Finally, 8 bacterial biomarkers were selected in the prediction model for distinguishing RA and OA in a non-invasive manner. This can be subjected to further clinical validation to develop new strategies for diagnosing RA and OA. Moreover, we need to further explore how bacteria can affect RA and OA, to help prevent and treat RA and OA.

## Methods

### Patients and Samples

This study was approved by the Institutional Review Board of Shandong Normal University and Shandong Academy of Medical Sciences. Written informed consents and questionnaire data sheets were obtained from all subjects who visited the Department of Orthopedics, Qianfoshan Hospital Affiliated to Shandong University, and Guoyitang Hospital. They agreed to serve as sample donors, in compliance with national legislation and the Code of Ethical Principles for Medical Research Involving Human Subjects of the World Medical Association (Declaration of Helsinki). All methods and experimental protocols in this study were performed in accordance with relevant guidelines and standard operating procedures. Saliva samples were collected using standard methods^35^: 5~10 mL of unstimulated whole saliva was collected in a 50 mL sterile tube from each subject, between 9~10 a.m. (collection time not more than 30 minutes). All subjects were asked to refrain from drinking, eating and hygiene-related procedures for at least 2 hours prior to collection, and all patients with apparent oral problems (periodontal disease *et al*.) were excluded. Samples were immediately frozen and stored at −80 °C. In all, 110 patients with RA who fulfilled the 2010 revised ACR/EULAR classification criteria were included^36^. 67 patients with OA met the 1995 revised ACR classification criteria^37^, and 155 healthy subjects served as controls (Table 1). The full list of sample information is available in the supplementary data (Table S1).

**Table 1:** Characteristics of all subjects.

	Healthy Subjects (n = 155)	RA (n = 110)	OA (n = 67)	*P*-values
Age (mean ± SD)	49.96 ± 11.17	56.65 ± 11.36	57.79 ± 9.712	*P* < 0.01
Gender (M/F)	80/75	20/90	21/46	*P* < 0.001

### DNA Extraction and 16S rRNA Gene Sequencing

For each sample, 1.5 ml of saliva was used for DNA extraction. The saliva was centrifuged at 12,000 × g for 15 min at 4 °C,and then the precipitation was used for DNA extraction (Qiagen DNeasy Blood & Tissue Kit). Quantitation of DNA was measured by Nanodrop 2000 (Thermo Scientific). To generate 16S rRNA gene amplicons, in a 50 ul reaction, typically 50 ng of DNA was used as a template, with 0.4 uM of V1–V2 barcoded primers, targeting 27 F and 355 R of the bacterial 16S rRNA gene (5′ AGAGTTTGATCMTGGCTCAG3′ and 5′ GCTGCCTCCCGTAGGAGT3′). DNA was purified with QIAquick PCR Purification Kit (Qiagen) and PCR purification procedure. All amplicons were quantified and pooled to equalize concentrations for sequencing, using HiSeq 2500 (Illumina).

### 16S rRNA Gene Sequence Analysis

The 16S rRNA gene sequence paired-end data set was joined and quality filtered using the FLASH method, described by Magoč and Salzberg^38^. All sequence analysis was provided in the Quantitative Insights Into Microbial Ecology (QIIME, version 1.9.1) software suite^39^, according to the QIIME tutorial (http://qiime.org/) with some modifications. Chimeric sequences were removed using usearch61^40^ with *de novo* models. Sequences were clustered against the 2013 Greengenes (13_8 release) ribosomal database’s 97% reference data set (http://greengenes.secondgenome.com/downloads). Sequences that did not match any entries in this reference were subsequently clustered into *de novo* OTUs at 97% similarity with UCLUST. A taxonomy was assigned to all OTUs using RDP classifier within QIIME and the Greengenes reference data set. Rarefaction and rank abundance curves were calculated from OTU tables using alpha diversity and rank abundance scripts within the QIIME pipeline. The hierarchical clustering based on population profiles of most common and abundant taxa was performed using UPGMA clustering (Unweighted Pair Group Method with Arithmetic mean, also known as average linkage), on the distance matrix of OTU abundance. This resulted in a Newick-formatted tree, which was obtained using the QIIME package.

### Statistical Analysis

To account for any bias caused by uneven sequencing depth, the least number of sequences present in any given sample from a sample category was selected randomly. Prior to calculating community-wide dissimilarity measures (α-diversity and β-diversity), we rarefied the OTU table to a sequencing depth of 40000 per sample for both diversity analyses. All PCoA were based on weighted UniFrac distances using evenly sampled OTU abundances. The prediction of the functional composition of a metagenome, using marker gene data and a database of reference genomes was done with PICRUSt as described by Langille *et al*.^41^. The graphical representation of the results was done with STAMP and the calculation of *P* values was done using Welch’s t-test (*P* values were corrected for Benjamini-Hochberg FDR).

### The ROC curves

The Support Vector Machine (SVM) classifier from R package e1071 was adopted for classification analysis of the selected biomarkers. Five-fold cross-validation was used to evaluate the performance of the prediction model. The ROC curves as well as the AUC value was calculated using the ROCR R package.

## Electronic supplementary material

Supplement Materials Table

Supplement Materials Figure

## Data Availability

The data sets generated during and/or analyzed during the current study are available with the corresponding author, on reasonable request.
