# Supplementary material for: Variations in oral microbiome profiles in rheumatoid arthritis and osteoarthritis with potential biomarkers for arthritis screening
Source: Sci Rep. 2018 Nov 20;8:17126. doi: 10.1038/s41598-018-35473-6 (PMC6244360; doi:10.1038/s41598-018-35473-6)
Supplement: Supplementary file 2 — Supplement Materials Figure [file 41598_2018_35473_MOESM2_ESM.pdf]

Supplementary Information for "Variations in oral microbiome profiles in rheumatoid arthritis and osteoarthritis with potential biomarkers for arthritis screening"

Bin Chen [1, #], Yan Zhao [1, 2, #], Shufeng Li [3], Lanxiu Yang [4], Haiying Wang [1], Tao Wang [2], Bin Shi [2], Zhongtao Gai [8], Xueyuan Heng [9], Chunling Zhang [7, *], Junjie Yang [5, *], Lei Zhang [1, 6, 7, 8, 9, 10 *]

[1] College of Life Science, Shandong Normal University, Jinan 250014, China

[2] Shandong Medicinal and Biotechnology Centre, Shandong Academy of Medical Sciences, Jinan 250062, China

[3] Department of Orthopedics, Qianfoshan Hospital Affiliated to Shandong University, Jinan 250014, China

[4] Guoyitang Hospital, Jinan 250000, China

[5] College of Life Science, Qilu Normal University, Jinan 250200, China

[6] Shandong Institutes for Food and Drug Control, Jinan 250101, China.

[7] Qingdao Human Microbiome Center, The Affiliated Central Hospital of Qingdao University, Siliu South Road 127, Qingdao, Shandong Province, 266042, China

[8] Shandong Children's Microbiome Center, Qilu Children's Hospital of Shandong University, Jinan, Shandong Province, 250022, China

[9] Microbiological Laboratory; Department of Infection Management; Department of Neurosurgery, Lin Yi People's Hospital, Linyi, Shandong Province, 276003, China

[10] Beijing Advanced Innovation Center for Big Data-Based Precision Medicine, School of Chemistry and Environment, Beihang University, Beijing 100191, China

[#] These authors contributed equally to this work

*Correspondence and requests for materials should be addressed to Lei Zhang (microbiome@foxmail.com), Junjie Yang (microbiota@foxmail.com), or Chunling Zhang (qdzcl2011@163.com)

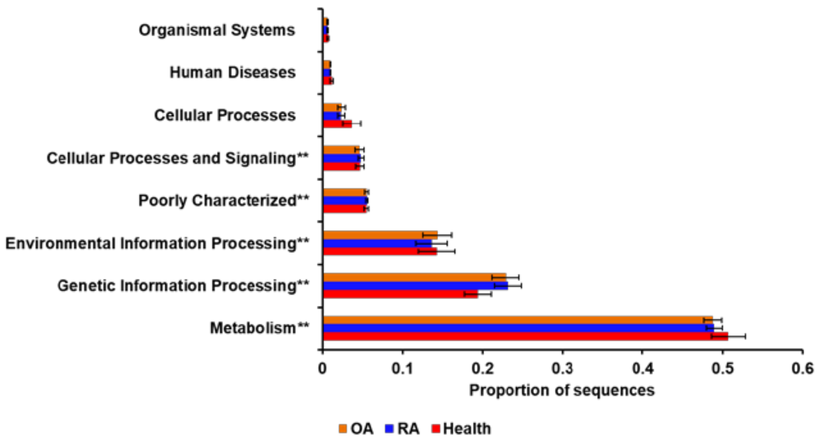

Figure S1 The KEGG pathways in RA, OA and Healthy control (level 1). The significantly different KEGG pathways in RA, OA and healthy control were marked with **($P< 0.01$).

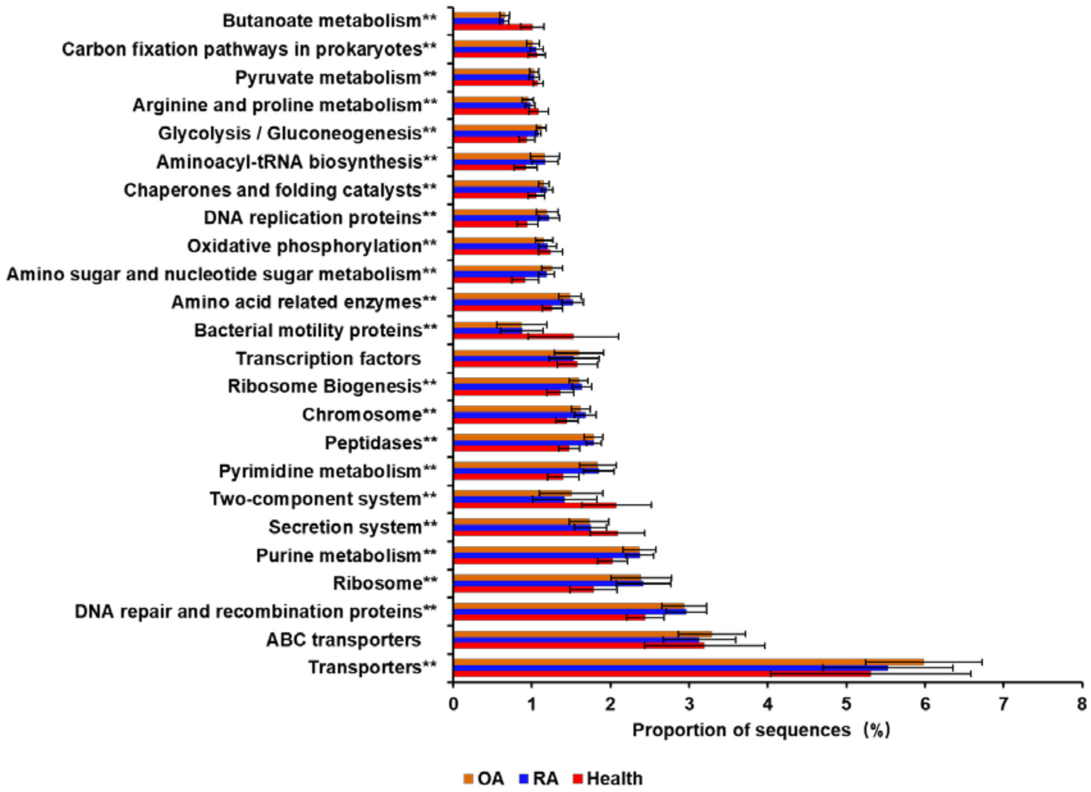

Figure S2 The KEGG pathways in RA, OA and Healthy control. Only show the 24 major KEGG pathways in level 3. The significantly different KEGG pathways in RA, OA and healthy control were marked with **($P< 0.01$).
